# Effect of room temperature transport vials on DNA quality and phylogenetic composition of faecal microbiota of elderly adults and infants

**DOI:** 10.1186/s40168-016-0164-3

**Published:** 2016-05-10

**Authors:** Cian J. Hill, Jillian R. M. Brown, Denise B. Lynch, Ian B. Jeffery, C. Anthony Ryan, R. Paul Ross, Catherine Stanton, Paul W. O’Toole

**Affiliations:** School of Microbiology, University College Cork, Cork, Ireland; Alimentary Pharmabiotic Centre, University College Cork, Cork, Ireland; Department of Neonatology, Cork University Maternity Hospital, Wilton, Cork, Ireland; Teagasc Food Research Centre, Moorepark, Fermoy, Cork, Ireland

**Keywords:** Microbiome, Microbiota, Storage, Methodology, Infant, Elderly

## Abstract

**Background:**

Alterations in intestinal microbiota have been correlated with a growing number of diseases. Investigating the faecal microbiota is widely used as a non-invasive and ethically simple proxy for intestinal biopsies. There is an urgent need for collection and transport media that would allow faecal sampling at distance from the processing laboratory, obviating the need for same-day DNA extraction recommended by previous studies of freezing and processing methods for stool. We compared the faecal bacterial DNA quality and apparent phylogenetic composition derived using a commercial kit for stool storage and transport (DNA Genotek OMNIgene GUT) with that of freshly extracted samples, 22 from infants and 20 from older adults.

**Results:**

Use of the storage vials increased the quality of extracted bacterial DNA by reduction of DNA shearing. When infant and elderly datasets were examined separately, no differences in microbiota composition were observed due to storage. When the two datasets were combined, there was a difference according to a Wilcoxon test in the relative proportions of *Faecalibacterium*, *Sporobacter*, *Clostridium XVIII*, and *Clostridium XlVa* after 1 week’s storage compared to immediately extracted samples. After 2 weeks’ storage, *Bacteroides* abundance was also significantly different, showing an apparent increase from week 1 to week 2.

The microbiota composition of infant samples was more affected than that of elderly samples by storage, with significantly higher Spearman distances between paired freshly extracted and stored samples (*p* < 0.001). When the microbiota profiles were analysed at the operational taxonomic unit (OTU) level, three infant datasets in the study did not cluster together, while only one elderly dataset did not. The lower microbiota diversity of the infant gut microbiota compared to the elderly gut microbiota (*p* < 0.001) means that any alteration in the infant datasets has a proportionally larger effect.

**Conclusions:**

The commercial storage vials appear to be suitable for high diversity microbiota samples, but may be less appropriate for lower diversity samples. Differences between fresh and stored samples mean that where storage is unavoidable, a consistent storage regime should be used. We would recommend extraction ideally within the first week of storage.

**Electronic supplementary material:**

The online version of this article (doi:10.1186/s40168-016-0164-3) contains supplementary material, which is available to authorized users.

## Background

The study of the human gut microbiota is a dynamic and rapidly expanding area of research as alterations in this complex ecosystem has been correlated with a wide range of health effects on the host, including obesity [1, 2], behaviour [3] and the immune system [4], in particular autoimmune diseases [5]. The need to separate correlation from causation has intensified the need for intervention studies in larger cohorts and multi-centre trials to account for other potential confounders [6, 7]. In recent years, the technical preparation of faecal samples has been shown to be an important issue for microbiota analysis [8, 9]. One important consideration when designing a gut microbiota study is to decide on the method that will be used to store the samples. Study design may not allow for immediate processing of fresh samples due to either workflow issues or geographical distance from sample collection centres to the processing laboratory. Depending on study design, subjects may report to labs or clinics for sample provision. It may also be necessary to collect fresh samples from subjects’ places of residence, which can be challenging for logistical reasons. The microbiome field therefore urgently requires a sampling method which is cost effective, easily applied outside of a clinical environment, and which produces an accurate representation of the microbiota composition without loss of any taxa [10].

A number of studies have therefore investigated the effect of different storage conditions on the apparent microbiota composition [11–14], including a recent study of the effect of three storage methods on the microbiota, freezing and fixation by RNAlater or ethanol [10]. The overall consensus from these studies indicates that processing freshly collected samples remains the gold standard where possible, but that a freeze-thaw cycle does not significantly alter apparent microbiota composition. Following storage, the method of extraction is very important [11, 13, 15, 16]. It is vital to employ a method which ensures that as much of the microbial DNA as possible is extracted from a sample, in particular gram-negative organisms and some Firmicutes which are difficult to lyse. Numerous studies have also determined the optimal set of primers to amplify ribosomal RNA gene regions in an unbiased manner [17–22]. The vast majority of projects use one or multiple regions of the 16S rRNA gene, although other genes have been proposed such as the gene for the heat shock protein, Cpn60 [18]. Each of these steps has the potential to introduce bias in apparent microbiota composition and must therefore be carefully managed. This is a major challenge when studying the microbiota or when comparing microbiota studies.

The OMNIgene GUT kit, from DNA Genotek (Ottawa, Canada) aims to eliminate a freeze-thaw step when temporarily storing or transporting stool samples for microbiota analysis. The product consists of a tube with a metal ball and stabilisation buffer. Once a faecal sample is placed in the tube and homogenised, it remains stable at room temperature. It thus offers advantages over traditional methods such as freezing samples by removing the need for a donor to freeze their sample at home, and it reduces the expense of transferring samples with ice packs or on dry ice. The product is designed to maintain a stable bacterial profile until and during transport to the laboratory for processing. We tested the ability of the OMNIgene GUT kit to stabilise faecal microbial DNA after both 1 and 2 weeks’ storage at room temperature in the provided tubes. We compared the microbiota composition after storage to the microbiota composition derived from the same stool samples processed immediately upon arrival at the laboratory, after transport at 4 °C. The data shows efficient maintenance of the microbiota composition for high-diversity microbiota samples, and some alterations of abundance for low diversity infant samples.

## Results

In order to determine how well the OMNIgene kit preserved DNA, we compared DNA extraction under the following conditions: fresh and after both 1 and 2 weeks’ incubation at room temperature in the storage vials provided in the kit—as per instructions for use; 22 infants and 20 elderly subjects were examined under these conditions. One elderly sample was subsequently removed due to low read numbers (see the “Methods” section). Additionally, the kit was compared to other commonly used storage conditions on a further subset of four subjects.

### DNA extraction

**Fig. 1:**
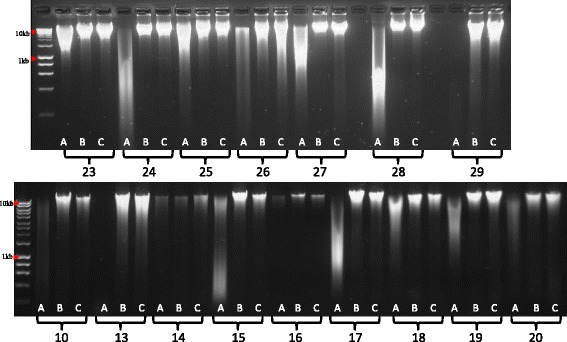
Agarose gel electrophoresis of elderly (*top*) and infant (*bottom*) samples after extraction. *A*, fresh; *B*, 1 week’s storage; *C*, 2 weeks’ storage. *Numbers* indicate subject IDs

Total faecal microbial DNA was extracted in each instance by a repeat bead beating (RBB) method (described in the “Methods” section), the major disadvantage of which is DNA shearing. After storage in the stabilising buffer, the technical steps involved in the RBB extraction were greatly improved. The separation between supernatant and waste pellet at all stages was better defined, and the final nucleic acid pellets were smaller, cleaner, and easier to re-suspend. Furthermore, electrophoretic analysis showed the DNA to be protected from shearing (Fig. 1), and the ratio of absorbance values at 260 nm:280 nm were improved (data not shown). The V4–V5 region amplicons from all samples were subjected to Illumina sequencing on a MiSeq instrument (2 × 300 paired end reads; average 74,173 reads per sample).

### Infant samples

**Fig. 2:**
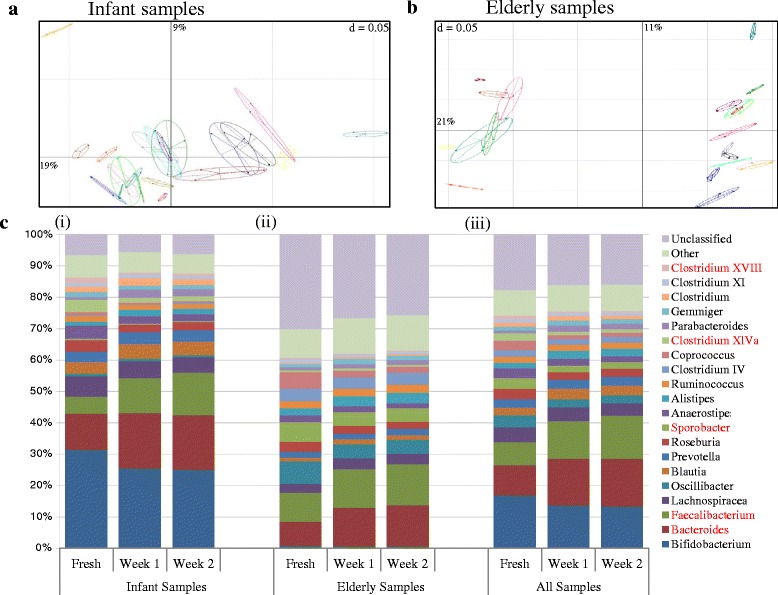
The effect of storage in DNA Genotek tubes for 1 and 2 weeks. **a** PCoA generated from a Spearman distance matrix, faecal samples from infant subjects, grouped by subject. **b** PCoA generated from Spearman distance matrix, showing elderly subject faecal samples. **c** Aggregate microbiota composition for (*i*) infant, (*ii*) elderly and (*iii*) all samples. The genera significantly altered, as according to the Wilcoxon signed-rank test, in analysis of all samples after storage for 2 weeks are indicated by *red text*

There was no detectable difference in the abundance profiles at genus level for any of the infant sample datasets comparing freshly processed samples and those prepared after either 1 or 2 weeks’ storage, despite the individual infant microbiota datasets appearing variable on a principal coordinate analysis (PCoA) graph (Fig. 2a). Examination of the aggregated compositions of all infant samples combined showed no significant difference at genus level between the relative abundance of any genera at time zero and after 2 weeks’ storage (Fig. 2c(i)). There was a trend towards higher *Faecalibacterium* abundance and a diminution of *Clostridium XVIII*, but the adjusted *p* value for the difference was not significant. There was a trend for some other bacterial genera to change abundance, specifically a reduction in *Bifidobacterium* in infant samples, but the magnitude of change did not reach statistical significance (data not shown).

### Elderly subjects

Similarly, we found that the derived faecal microbiota composition of the elderly subjects was minimally affected by storage in the stabilising buffer, as shown in the PCoA plot (Fig. 2b) and genus-level microbiota composition (Fig. 2c(ii)). The freshly extracted samples and the storage replicates grouped closely together. There was no significant change in the relative abundance of any genera in fresh processed samples and samples stored in the stabilisation buffer for 1 week when examining them at a population level using DeSeq2.

### Infant and elderly samples combined

When the aggregate microbiota across each respective time point was determined and compared, some significant differences were observed when examined by Wilcoxon signed-rank test (Fig. 2c(iii)). There was a significant change in the relative abundance of *Faecalibacterium* (*p* < 0.001), *Sporobacter* (*p* < 0.01), *Clostridium XVIII*, and *Clostridium XlVa* (*p* < 0.05) after 1 week of storage in the tubes. After 2 weeks’ storage, these genera remained significantly different from fresh and now also with a significant increase in *Bacteroides* abundance (*p* < 0.05). This suggests that the microbiota composition is relatively consistent after storage in the stabilising buffer at room temperature, but that the stability is reduced over time due to subtle changes which occur when incubated in the buffer. The genus *Bifidobacterium* displayed lower proportions after storage but due to the higher inter-individual variation of the level of *Bifidobacterium* in the infants, the difference was not statistically significant when analysed by Wilcoxon signed-rank test. In order to examine the dataset by further statistical test methods, another statistical method was utilised, a paired DESeq test. In contrast to the Wilcoxon signed-rank test, there was a much larger number of genera displaying different relative abundance between freshly extracted samples and data derived from both 1 and 2 weeks’ of storage (Additional file 1: Table S1). This suggests that subtle but significant changes occur in the stabilising buffer compared to fresh extraction. It is important to note that there were no significant differences between week 1 and 2 microbiota composition, regardless of the statistical test applied. This indicates that the tubes are consistent in the profiles returned once stored, despite returning subtle changes in absolute microbiota composition.

### Diversity of samples

**Fig. 3:**
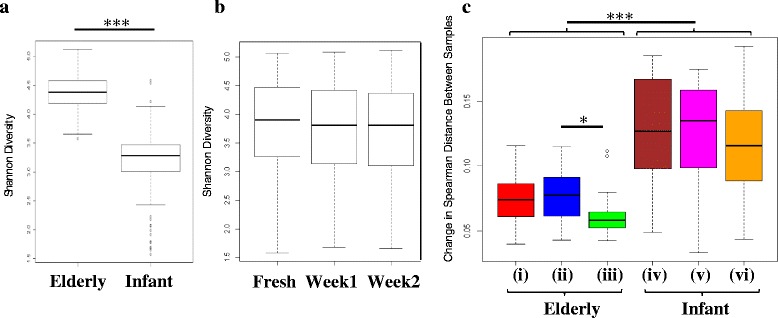
Elderly subjects have higher diversity than infant subjects and are less variable after storage. **a** Shannon diversity of infant and elderly samples. **b** Shannon diversity values after storage across infant and elderly subject samples combined. **c** Higher variabilty of infant subjects demonstrated by increase in the absolute Spearman distances between samples. (*i*–*iii*) elderly samples and (*iii*–*vi*) infant samples. (*i*) and (*iv*) fresh vs 1 week’s storage; (*ii*) and (*v*) fresh vs 2 weeks’ storage; (*iii*) and (*vi*) 1 week’s storage vs 2 weeks’ storage

The microbiota diversity of the infant samples, as measured by the Shannon index, was much lower than that of the elderly subjects (Fig. 3a, *p* < 0.001). Storage of the samples had no effect on this diversity after either 1 or 2 weeks (Fig. 3b). The apparent infant microbiota composition exhibited significantly higher variation than that of elderly subjects’ samples, reflected in the Spearman distance between storage time points (Fig. 3c). The difference between stored samples at week 1 and week 2 in the elderly subjects (Fig. 3c(iii)) was significantly lower than the difference between fresh and either 1 week (Fig. 3c(i)) or 2 weeks’ (Fig. 3c(ii)) storage. The difference was consistently higher under all storage conditions in the infant samples. This demonstrated that individual infant samples tended to display greater difference between the microbiota composition of freshly extracted samples and those stored before extraction. This suggested that the lower diversity of the infant gut microbiota resulted in a higher susceptibility to non-specific changes in the profile of the gut microbiota after storage. The individual infant and elderly microbiota composition data are presented in Additional file 2: Figures S1 and Additional file 3: Figure S2, respectively.

### Samples cluster with other samples from the same subject after storage

To further investigate the differences in apparent microbiota composition between stored and freshly processed samples, we generated a dendrogram using average clustering on a Spearman distance matrix (Additional file 4: Figure S3). Each of the three samples from all 19 elderly subjects clustered together, with one exception, demonstrating that they were more similar to one another than any other sample. In the case of subject 41, the 2-week sample extraction did not group with the freshly processed and week 1 sample. There was no obvious reason from the taxonomic classification of the samples as to why they did not cluster together (Additional file 3: Figure S2). Of the 22 infant samples, three (14 %) of the microbiota composition datasets did not cluster together across all three time points. The infant samples that did not cluster with themselves, but rather with samples from other subjects, did not retain a consistent bacterial profile after storage (Additional file 2: Figure S1).

### OMNIgene gut storage vials produce comparable microbiota data to common storage methods

**Fig. 4:**
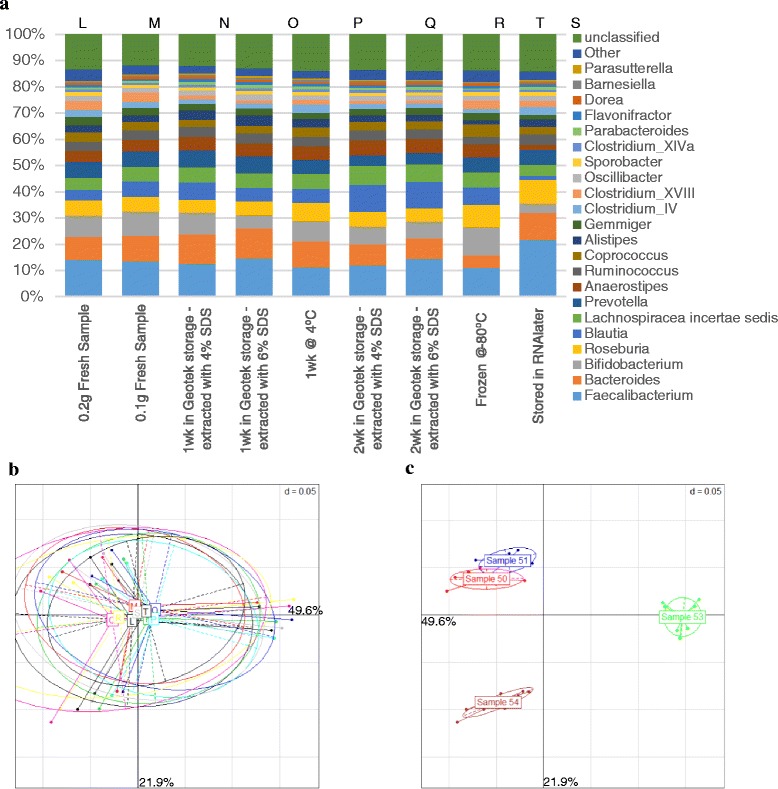
Effect of storage conditions on the microbiota composition of human faecal samples. **a** The aggregated microbiota composition of four subjects with indicated treatment conditions—no significant differences were observed at genus level between any of the different conditions of extraction or storage after analysis by Wilcoxon signed-rank test or DESeq. **b** PCoA of samples, grouped by treatment conditions. No significant differences observed at OTU level when examined by PerMANOVA analysis of the Spearman distance matrix. Condition labelled L–T—as displayed above genus level *bar charts* in part A. **c** PCoA of samples, grouped by subject number. Significant differences were observed at OTU level when examined by PerMANOVA analysis of the Spearman distance matrix. *p* < 0.001 between all subjects

To investigate if the OMNIgene gut kit vials performed comparably to commonly used storage methods, four samples (two infant, two elderly) were extracted under a range of different conditions (Fig. 4). To examine if there was any effect of dilution of the sodium dodecyl sulfate (SDS) concentration in the RBB lysis buffer due to the storage buffer, samples were stored as previously in the vials but extracted with 6 % SDS. This produced a final working concentration of 4 % SDS; 0.1 g of stool was also extracted fresh to examine if the amount of starting material was a factor. Other sample storage conditions tested were storage at 4 °C for a week, frozen at −80 °C, and fixed overnight in RNAlater for 24 h before freezing at −80 °C (as per [10]), as described in the “Methods” section. No significant effect upon the microbiota composition was apparent for any of the storage techniques at operational taxonomic unit (OTU), genus, or phylum levels after analysis by DESeq (Fig. 4). Shannon diversity analysis revealed no significant effect of any of the storage methods tested on these four samples (data not shown). The profiles are consistent across the numerous different experimental conditions; however, it should also be noted that the small sample size reduces the statistical power of the statistical models used.

One infant sample was extracted under a number of these different conditions (see the “Methods” section), with the extracted bacterial DNA from the different treatments amplified in duplicate using two different amplification barcodes. No differences in microbiota profile were observed (Additional file 5: Figure S5), with all 18 microbiota datasets from the subject clustered together and 8 of the 9 PCR duplicate pairs grouped together on the dendrogram (Additional file 4: Figure S3), showing negligible bias due to PCR amplification.

## Discussion

The OMNIgene gut kit offers an apparent solution to the problem of sample collection and processing, a problem recognized in the literature [10]. The extraction method used for this study was the RBB method described by Yu and Morrison [24], with some minor adjustments (see the “Methods”). A major disadvantage of the method is shearing of DNA. Storage of sample in the stabilising buffer greatly reduced shearing of the microbial DNA, and this higher quality DNA resulted in greater PCR efficiency.

We have demonstrated here the performance of the storage tubes in preserving the microbial profile of stool in the stabilising buffer. Upon examination of the data from the 41 subjects together, it was observed that after 1 week of storage, relative abundances of *Faecalibacterium*, *Sporobacter*, *Clostridium XVIII*, and *Clostridium XlVa* were significantly different when compared to the freshly extracted samples. There was also a non-significant alteration in the abundances of *Bacteroides* after 1 week’s storage when compared to fresh extraction, a difference which becomes statistically significant after 2 weeks. This indicates a slight increase in the instability after increased duration of storage when examined by paired Wilcoxon test. However, DESeq analysis, which uses a model based on the negative binomial distribution, detected numerous subtle differences at genus level in the microbiota composition of fresh and stored samples. Both analyses detected changes associated with differential storage; however, these changes are smaller than the variance between subjects and thus do not affect the Beta diversity of the microbiota. No significant alterations were observed in the microbiota once the samples were stored using the kit, showing that individual microbial profiles remained consistent. This is important as two studies using two different approaches (fresh versus room temperature transport vials) will identify the same taxonomic differences and trends in the microbiota between groups. However within a study, an investigator should not mix the two storage approaches. Even though there are no significant microbiota alterations between week 1 and week 2, we cannot rule out minor changes over time and so although it may have a minimal impact, steps should be taken to record the length of time in storage to ensure that it is not a confounding variable. It is possible that either bacterial growth may occur in the environment of the stabilising buffer, or more likely, differential lysis and DNA degradation accompanies storage in the buffer.

As is well established [6], infant samples harboured lower diversity microbiota than adults. This lower diversity means that any variation in microbiota composition caused by storage of the samples in the stabilising buffer would have a proportionally greater effect on the infant microbiota composition. In an attempt to account for any variation that may be observed between fresh extraction and storage in the stabilising buffer, one infant sample was extracted under nine different conditions. The individual variation observed in subjects may simply be due to the section of the stool sample used (i.e. taking the sample for the storage tube and the fresh samples from different parts of the stool). It has been shown in the past that repeated sampling of a different physical site from the same stool sample can lead to some variations in microbiota composition [12, 23]. However, a technical replicate of one fresh extracted elderly subject’s sample (subject 24, Additional file 3: Figure S2) showed high similarity despite extraction having been carried out on two separate 0.2-g stool samples. Separate 0.1 g and 0.2 g extractions were also extracted for four subjects, showing high reproducibility (Additional file 6: Figure S4). In addition to potential sampling bias, bias could have been incurred by PCR amplification, although the duplicated PCR amplification of all nine samples from subject 22 (Additional file 5: Figure S5) shows that this explanation of the observed individual variation is improbable.

When comparing the DNA Genotek OMNIgene gut kit to currently commonly used storage techniques, it demonstrates comparable efficacy in maintaining the composition of the gut microbiota as sampled from stool samples (Fig. 4). The profiles remain taxonomically consistent in our method comparison, but it should be noted that more subject numbers with increased statistical power may be required to discern if there are alterations in the gut microbiota due to these differential storage conditions. As most common alternative methods involve freezing, this commercially available kit is demonstrated to be a suitable alternative to patient freezing of stool samples for the study of the gut microbiota.

## Conclusions

The DNA Genotek OMNIgene gut kit offers a solution to the current common practice of patients freezing samples in home freezers, which is a barrier to participation in studies for many people. It could also potentially reduce the cost of transporting frozen samples, particularly if they are transferred on dry ice which is expensive, can cause burns to the skin if handled improperly, and asphyxiation. Despite some individual faecal samples showing differences in microbiota composition between freshly extracted and stored samples, the Genotek OMNIgene gut kit may offer a method for consistently stabilising bacterial DNA without requiring freezing. It also protects microbial DNA from shearing during extraction, which could have benefits for other downstream processes that require high molecular weight DNA, i.e. shotgun sequencing. When using the OMNIgene gut kits, we would recommend extraction within 1 week if possible as the aggregated microbiota profile seems to undergo some genera-specific alterations due to incubation in the stabilising buffer, and these alterations may increase over time. Any changes have a proportionally greater effect on low diversity samples. Since changes in the proportional abundance of genera were only found to be statistically significant when using the larger cohort comprising all subjects, this may indicate that further research with a greater number of subjects may identify other genera whose apparent abundances are affected by the storage conditions.

## Methods

We collected infant faecal samples from 22 subjects, ranging in age from 4 weeks to 2 years of age (median 2 years of age, interquartile range 6 months to 2 years old), and 20 elderly samples, from subjects ranging in age from 34 to 83 years of age (median 70 years old, interquartile range 68 to 75 years old) (Additional file 7: Table S2). One of the elderly donor samples was removed from the study due to low read numbers (334 reads) of the freshly processed sample, which was below the cut-off point of 20,000 reads per sample.

### Sample collection

Infant faecal samples were obtained from the INFANTMET cohort. Elderly subjects’ samples were collected as part of the ELDERMET study. These studies were approved by Clinical Research Ethics Committee of the Cork Teaching Hospitals. Subjects, or their mother in the case of the infant subjects, have signed forms consenting to the study. Samples were collected from individual’s home by a research nurse and immediately transferred to the lab on ice. Upon receiving the stool in the lab, DNA from 0.2 g was immediately extracted. Another 0.2 g portion was transferred to the DNA Genotek tube. This tube contains a stabilising liquid and a mixing ball. The stool sample was homogenised for 30 s by vigorous shaking. It was then stored at room temperature for extraction after 1 and 2 weeks, respectively.

### Extraction method

DNA was extracted from a single stool sample three times; 0.2 g of stool immediately at collection time without using the commercial collection kit and another 0.2 g of the same stool was placed into the OMNIgene gut kit tube containing a proprietary stabilising buffer, homogenised, and stored at room temperature. After 1 week’s storage at room temperature in the OMNIgene gut kit tube containing the stabilising buffer, 0.8 ml of this suspension was extracted. After a second week’s incubation at room temperature, another 0.8 ml of the stool/buffer suspension was extracted.

For the fresh extractions, microbial DNA was extracted from 0.2-g stool samples using the RBB method described by Yu and Morrison [24], with some modifications. In brief, 0.2 g of stool was incubated with 1 ml RBB lysis buffer (500 mM NaCl, 50 mM Tris-HCl, pH 8.0, 50 mM EDTA, and 4 % SDS) in a fresh 2-ml screw cap tube with 0.5 g sterile zirconia beads (1 3.0 mm bead, 0.1 g of 0.5 mm beads, and 0.3 g of 0.1 mm beads). It was homogenised via bead beating for 90 s (Mini-Beadbeater™, BioSpec Products, Bartlesville, OK, USA), with the samples cooled on ice for 60 s before another 90-s bead beating. Samples were incubated at 70 °C for 15 min to further lyse the cells. These samples were centrifuged, the supernatant removed, and these RBB steps were repeated with 0.3 ml of RBB lysis buffer. The supernatants were pooled and incubated with 350 ml of 7.5 M ammonium acetate (SIGMA). The DNA was precipitated by isopropanol, spun down into a nuclear pellet which was washed by 70 % ethanol. The pellet was allowed to dry and was re-suspended in TE buffer and treated with RNAse and Proteinase K. It was cleaned with QIAGEN buffers AW1 and AW2 and eluted in 200 μl of AE buffer (QIAamp DNA Stool Mini Kit, QIAGEN, UK). DNA was visualised on a 0.8 % agarose gel and quantified using the Nanodrop 1000 (Thermo Scientific, Ireland). Extracted DNA was stored at −80 °C.

For samples stored in the OMNIgene gut tube with stabilising buffer, 0.2 g of the stool sample is placed in the homogeniser and homogenised by shaking vigorously for 30 s, aided by a metal ball. This was done in parallel with the fresh extraction upon arrival of the stool sample to the laboratory. The proprietary stabilising buffer is designed to preserve the integrity of the microbiota without freezing and is designed to remain effective at room temperature. For the storage samples, 0.8 g of the homogenised sample in the stabilisation buffer was added to the 2-ml screw cap tubes containing zirconia beads, as per fresh extractions. Then, a further 0.8 ml of RBB lysis buffer was added. The extraction then proceeded as per the fresh sample RBB extraction protocol.

### Alternative storage conditions

Since the re-suspension of the stool in storage buffer resulted in additional liquid being added to the DNA extraction, this caused dilution of the concentration of SDS in the lysis buffer, which may have affected the extraction efficiency. To investigate this, we prepared faecal samples from four subjects under a variety of conditions. They were extracted using lysis buffer containing either 6 % (*w*/*v*) SDS or the normal 4 % SDS, as per the protocol above. This was to determine if the dilution of SDS by the stabilising buffer was influencing the results. The remainder of the sample in the OMNIgene tubes were also stored at room temperature and extractions were repeated after 1 and 2 weeks.

In order to compare to commonly used storage methods, we investigated the efficacy of a number of further storage conditions using these four subjects. We extracted 0.1 g stool fresh, without placing in the OMNIgene stabilising buffer, in addition to the regular 0.2 g RBB extraction from fresh sample. The stool sample was stored at 4 °C for a week and then extracted. A section of the stool sample was fixed in RNAlater for 24 h before freezing as per Franzosa et al. [10], and a further section was immediately frozen at −80 °C before extraction after 1 week.

For one infant sample (sample 22), the mother of the infant immediately placed 0.2 g of a stool sample in a storage tube after defecation. The remainder of this stool sample was transferred to the lab in a regular stool collection tube at 4 °C; 0.2 g of this sample was then transferred to a storage tube in the laboratory in order to examine if the microbiota was altered during transport. These two samples were then immediately extracted to investigate whether incubation with the OMNIgene stabilising buffer without storage for any length of time affected the composition of the microbiota. This was again done in duplicate with 6 and 4 % SDS buffer to act as a control. All samples from this infant were PCR amplified in duplicate to assess the effect of PCR bias between samples.

### 16 s rRNA primers

The bacterial primers used for PCR amplification were the V4–V5 region primers 520F (AYTGGGYDTAAAGNG) and 926R (CCGTCAATTYYTTTRAGTTT). Initial primers for Illumina sequencing contain the sequencing primer binding sites, forward or reverse 16S-specific primer, and a 10-nt in-line multiplexing identifier (MID). Dual separate MID were attached to both the ends of the PCR product (Additional file 8: Table S3).

The V4–V5 amplicons for Illumina sequencing were generated using a two-step amplification procedure. The first step reaction mix contained 50 μl BIO-X-ACT™ Short Mix (BIOLINE), 10 μl of 2 nM forward and reverse primers, 50 ng genomic DNA, and ddH_2_0 to give a final volume of 100 μl. Cycling conditions were an initial 95 °C, 5-min denaturation step; 30 cycles of 95 °C for 15 s, 42 °C for 15 s, and 72 °C for 30s; and a final 10-min extension at 72 °C. The products were purified using SPRIselect beads (Beckman Coulter, Indianapolis IN) as per manufacturer instructions, using a 0.9:1 volume ratio of beads to product. The purified PCR products were eluted in 40 μl of ddH_2_0. DNA quantity was assessed via Quant-iT™ PicoGreen® dsDNA Assay Kit (Invitrogen™). The samples were pooled in equimolar amounts and sent to the sequencing service of the University of Exeter for library preparation and Illumina MiSeq 2x300 bp paired end sequencing run. Nextflex Rapid library preparation was carried out by the company to attach bridge adaptors necessary for clustering.

### Bioinformatic pipeline

The obtained paired-end MiSeq sequences were joined using FLASH (Fast Length Adjustment of SHort reads to improve genome assemblies) programme [25]. The MIDs were extracted and sequences were split into individual samples and quality filtered using QIIME’s split_libraries_fastq.py, allowing for two ambiguous bases per sequence (Ns). Sequences were quality filtered using the USEARCH sequence analysis tool. In brief, sequences were filtered by length, single unique reads removed, and the remaining reads were clustered into OTUs. Chimeras were removed with UCHIME, using the GOLD reference database. The original input sequences were mapped onto the OTUs with 97 % similarity. An OTU table was subsequently generated using a perl script. All reads were classified to genus level by MOTHUR using the RDP reference database [26]. OTUs were classified from these, when >50 % of the reads agreed on a classification. Statistical calculations and outputs were performed in R statistical framework and Microsoft Excel. The returned read numbers varied greatly from 273,713 to 334 reads. To combat the influence on the number of sequences in a sample on diversity and other statistical tests, any sample under 10,000 sequences was discarded. This resulted in the loss of one sample (subject33 fresh sample) from the data set. Since this meant that only stored samples were available for this subject, it was removed from the analysis, leaving 19 elderly subjects in the study. The OTU table containing the remaining 139 samples was converted to a proportion OTU table to reduce any bias from increased read number of certain samples.

R statistical software package was used for all statistical analysis and visualisation. Software libraries used included made4, vegan, and DESeq2. The diversity index used was the Shannon index. PCoA based on the Spearman distance ((1 − Spearman correlation)/2) of relative abundances of OTUs was performed to determine clustering patterns among subjects. Differences between groups were tested for using PERMANOVA. To detect significant differences between genera at different time points, both the Wilcoxon signed-rank test and paired DESeq2 software package was used and the adjusted *p* value was used to test for significance. Proportional genus level charts were generated using R and visualised by Microsoft Excel.

## Additional files

Additional file 1: Table S1. Summary of the significantly altered genera after 1 and 2 weeks’ storage in the DNA Genotek OMNIgene gut kit as determined by paired DESeq analysis. Statistically significant increases in relative abundance are shown in red and significant reductions are shown in green. Both log fold change and adjusted *p* values are presented. (XLSX 13 kb) Additional file 2: Figure S1. Microbiota composition of each sample for every infant subject in the study. A = Fresh, B = 1 week’s storage, C = 2 weeks’ storage. Numbers indicate subject IDs. (PDF 120 kb) Additional file 3: Figure S2. Microbiota composition of each sample for every elderly subject in the study. A = Fresh, B = 1 week’s storage, C = 2 weeks’ storage. Numbers indicate subject IDs. Subject 33 was removed from the main study due to the fresh sample having insufficient read number for analysis. Subject 24 has two technical replicates for the fresh sample to demonstrate the reproducibility of RBB extraction method. (PDF 132 kb) Additional file 4: Figure S3. Majority of samples cluster by subject showing stability of microbiota composition after storage. A) Dendrogram using hierarchical clustering of a Spearman distance matrix, showing clustering of samples with their technical replicates. Subject 41 is highlighted as the only elderly subject where the samples did not cluster together B) Magnification to indicate infant subjects. Subjects 08, 09 and 16 are highlighted as subjects where samples do not cluster together. Infant subject 22 was extracted under nine difference conditions, and each condition was replicated by a second PCR. (PDF 245 kb) Additional file 5: Figure S5. In depth sampling of one stool sample from an infant subject, with different storage conditions and different concentrations of SDS in lysis buffer. A) 0.2 g of the sample incubated immediately in DNA Genotek storage tube by mother. Remainder of stool sample transferred to lab in regular tube. B) 0.2 g transferred to DNA Genotek storage tube in lab. C) 0.2 g sample extracted without storage tube. Samples extracted either immediately or after 1 week’s storage (w0 or w1, respectively) with 4 or 6 % SDS lysis buffer, as indicated, and amplified in duplicate ((i) and (ii)). (A) and (B) samples were stored at room temperature in storage tubes, while (C) was stored at 4 °C in a regular sample collection tube before w1 extraction. (PDF 1450 kb) Additional file 6: Figure S4. Microbiota composition of each sample of four subjects after all conditions of storage. L = Fresh extraction 0.2 g stool; M = Fresh extraction 0.1 g stool; N = storage for 1 week in DNA Genotek storage vial and extracted with 4 % SDS RBB lysis buffer; O = storage for 1 week in DNA Genotek storage vial and extracted with 6 % SDS RBB lysis buffer; P = storage in regular stool collection tube at 4 °C for 1 week; Q = storage for 2 weeks in DNA Genotek storage vial and extracted with 4 % SDS RBB lysis buffer; R = storage for 2 weeks in DNA Genotek storage vial and extracted with 6 % SDS RBB lysis buffer; S = Frozen at−80 °C for a week prior to extraction; T = 0.2 g of stool fixed overnight in 700 μl of RNAlater before freezing at−80 °C for a week. (PDF 54 kb) Additional file 7: Table S2. Subject details for each member in the study. Subjects 50, 51, 53, 54 were not part of the main study but were used for comparison of storage methods (Fig. 4). (XLSX 11 kb) Additional file 8: Table S3. Primers and barcode sequences used in the study. (XLSX 13 kb)

## Abbreviations

FLASH: fast length adjustment of short reads to improve genome assemblies
MID: multiplexing identifier
OTU: operational taxonomic unit
RBB: repeat bead beating
SDS: sodium dodecyl sulfate
