# Supplementary figures and images for: Effect of room temperature transport vials on DNA quality and phylogenetic composition of faecal microbiota of elderly adults and infants
Source: Microbiome. 2016 May 10;4:19. doi: 10.1186/s40168-016-0164-3 (PMC4862223; doi:10.1186/s40168-016-0164-3)

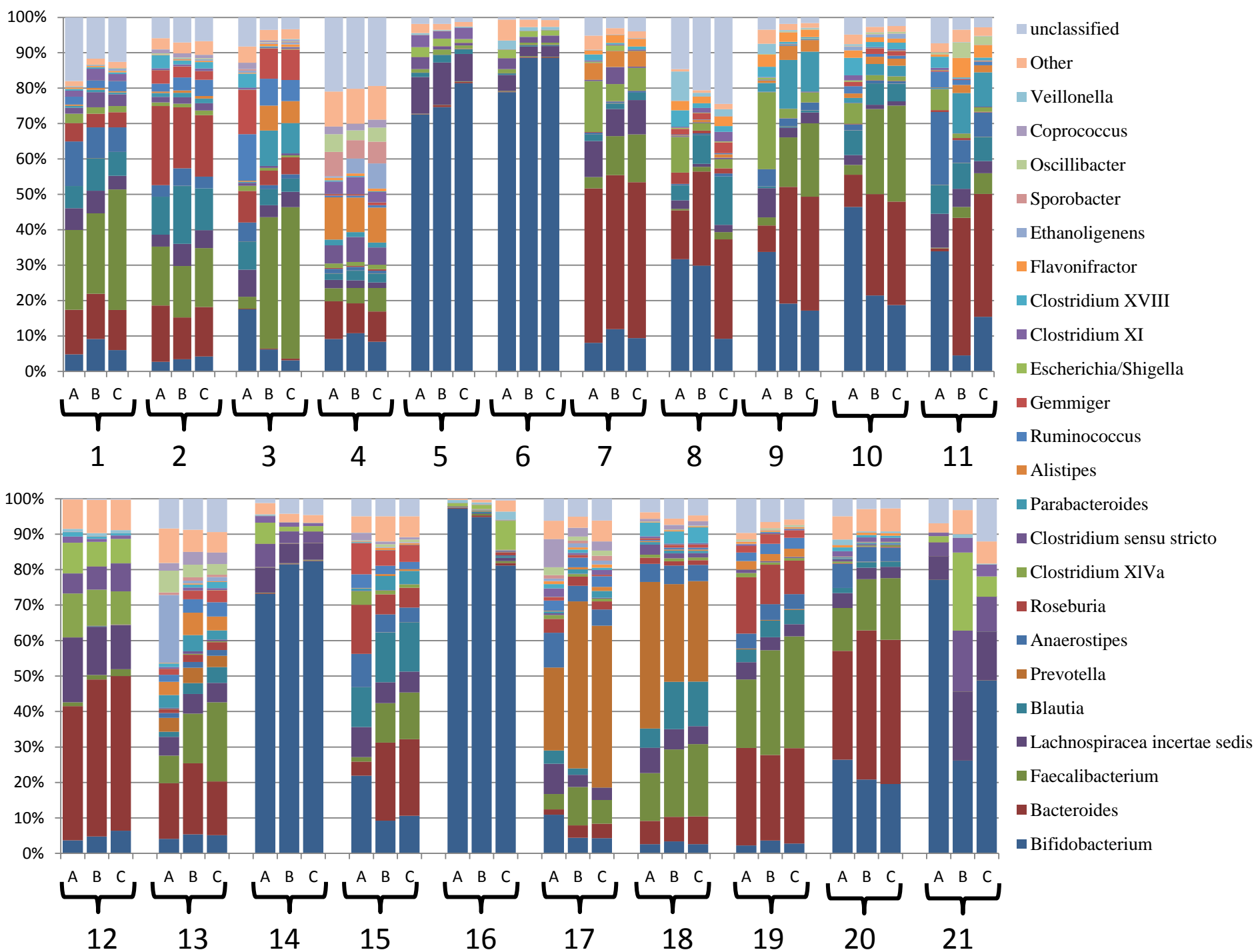

Supplement: Additional file 2: Figure S1. — Microbiota composition of each sample for every infant subject in the study. A = Fresh, B = 1 week’s storage, C = 2 weeks’ storage. Numbers indicate subject IDs. (PDF 120 kb) [file 40168_2016_164_MOESM2_ESM.pdf]

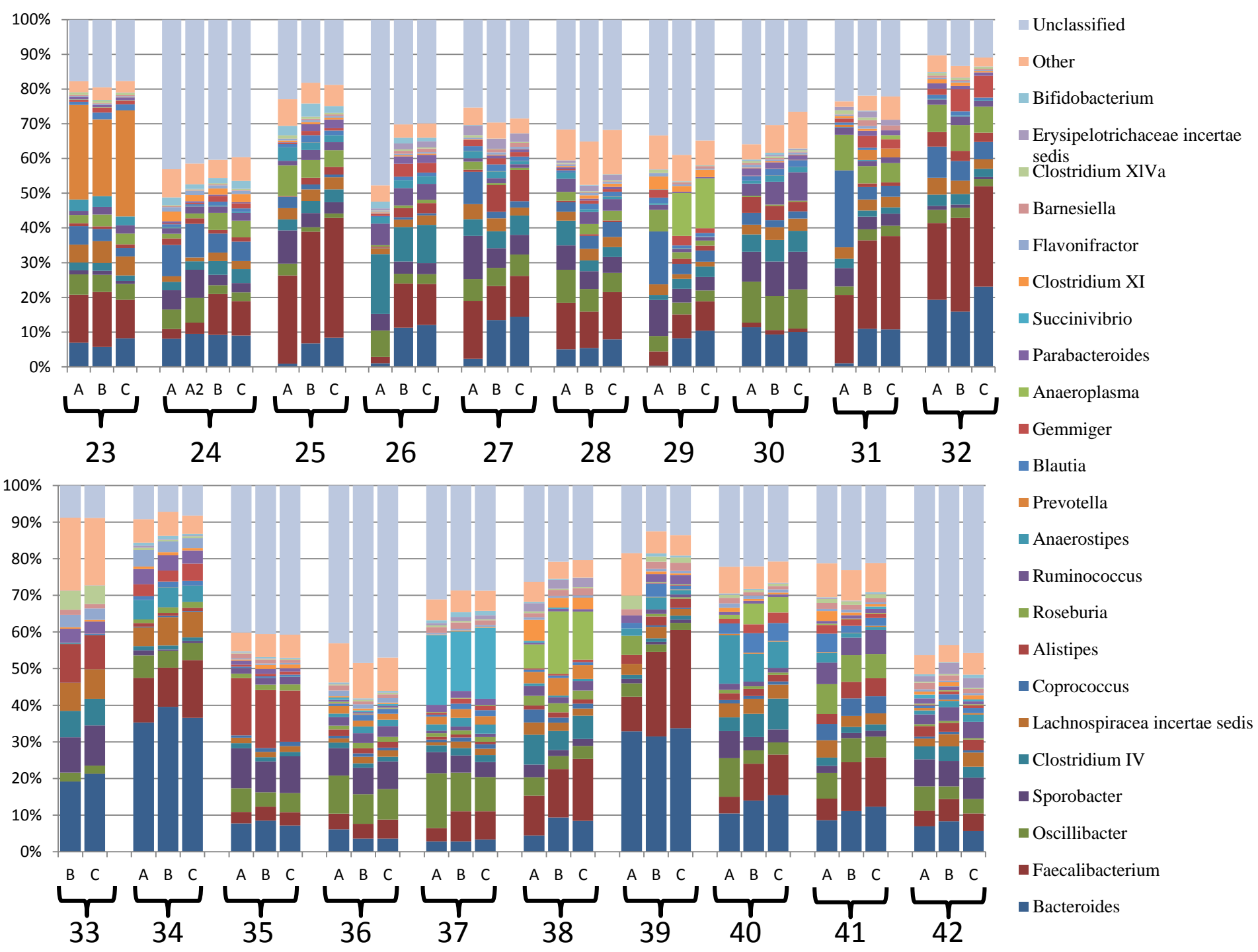

Supplement: Additional file 3: Figure S2. — Microbiota composition of each sample for every elderly subject in the study. A = Fresh, B = 1 week’s storage, C = 2 weeks’ storage. Numbers indicate subject IDs. Subject 33 was removed from the main study due to the fresh sample having insufficient read number for analysis. Subject 24 has two technical replicates for the fresh sample to demonstrate the reproducibility of RBB extraction method. (PDF 132 kb) [file 40168_2016_164_MOESM3_ESM.pdf]

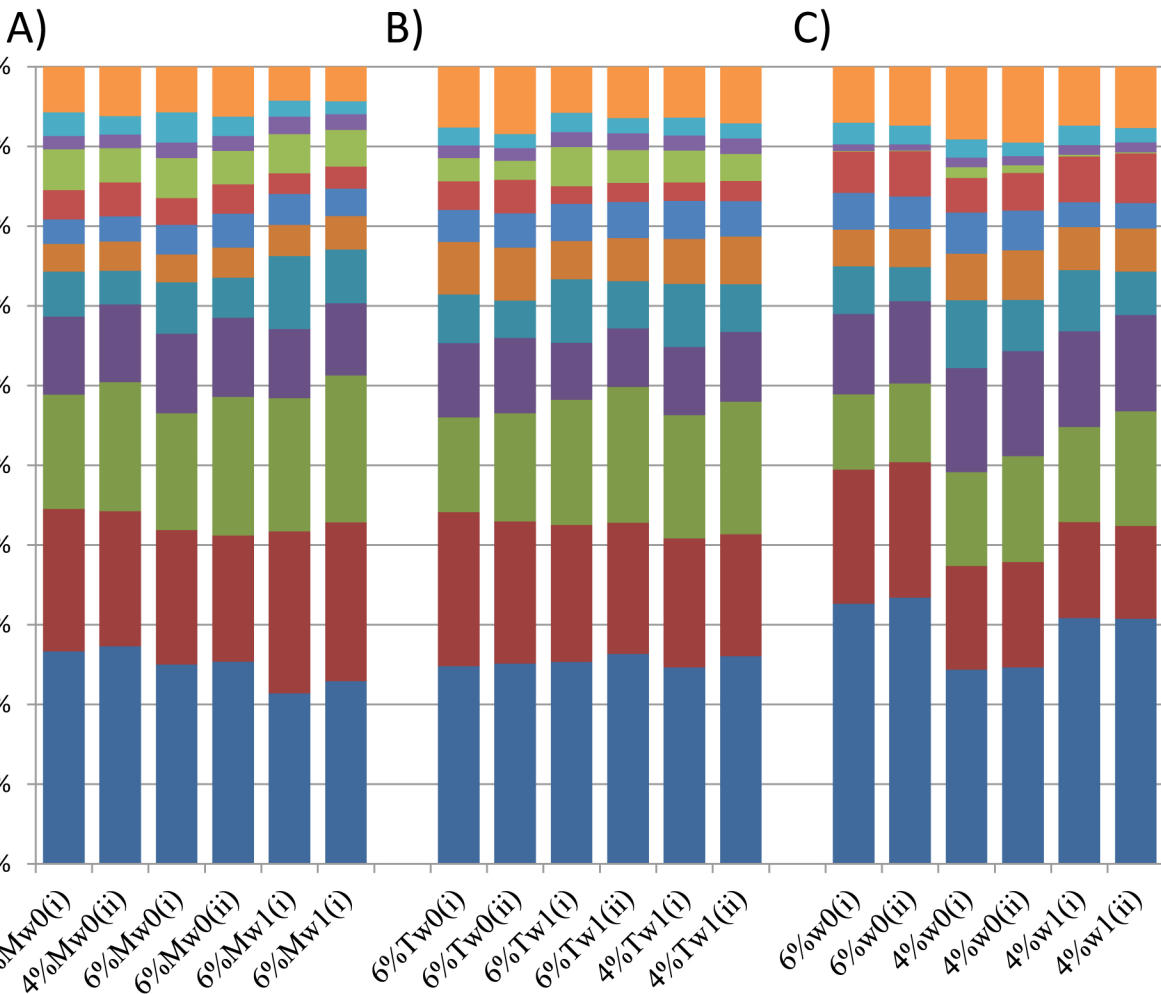

Supplement: Additional file 5: Figure S5. — In depth sampling of one stool sample from an infant subject, with different storage conditions and different concentrations of SDS in lysis buffer. A) 0.2 g of the sample incubated immediately in DNA Genotek storage tube by mother. Remainder of stool sample transferred to lab in regular tube. B) 0.2 g transferred to DNA Genotek storage tube in lab. C) 0.2 g sample extracted without storage tube. Samples extracted either immediately or after 1 week’s storage (w0 or w1, respectively) with 4 or 6 % SDS lysis buffer, as indicated, and amplified in duplicate ((i) and (ii)). (A) and (B) samples were stored at room temperature in storage tubes, while (C) was stored at 4 °C in a regular sample collection tube before w1 extraction. (PDF 1450 kb) [file 40168_2016_164_MOESM5_ESM.pdf]
