# Supplementary material for: Effect of room temperature transport vials on DNA quality and phylogenetic composition of faecal microbiota of elderly adults and infants
Source: Microbiome. 2016 May 10;4:19. doi: 10.1186/s40168-016-0164-3 (PMC4862223; doi:10.1186/s40168-016-0164-3)
Supplement: Additional file 6: Figure S4. — Microbiota composition of each sample of four subjects after all conditions of storage. L = Fresh extraction 0.2 g stool; M = Fresh extraction 0.1 g stool; N = storage for 1 week in DNA Genotek storage vial and extracted with 4 % SDS RBB lysis buffer; O = storage for 1 week in DNA Genotek storage vial and extracted with 6 % SDS RBB lysis buffer; P = storage in regular stool collection tube at 4 °C for 1 week; Q = storage for 2 weeks in DNA Genotek storage vial and extracted with 4 % SDS RBB lysis buffer; R = storage for 2 weeks in DNA Genotek storage vial and extracted with 6 % SDS RBB lysis buffer; S = Frozen at−80 °C for a week prior to extraction; T = 0.2 g of stool fixed overnight in 700 μl of RNAlater before freezing at−80 °C for a week. (PDF 54 kb) [file 40168_2016_164_MOESM6_ESM.pdf]

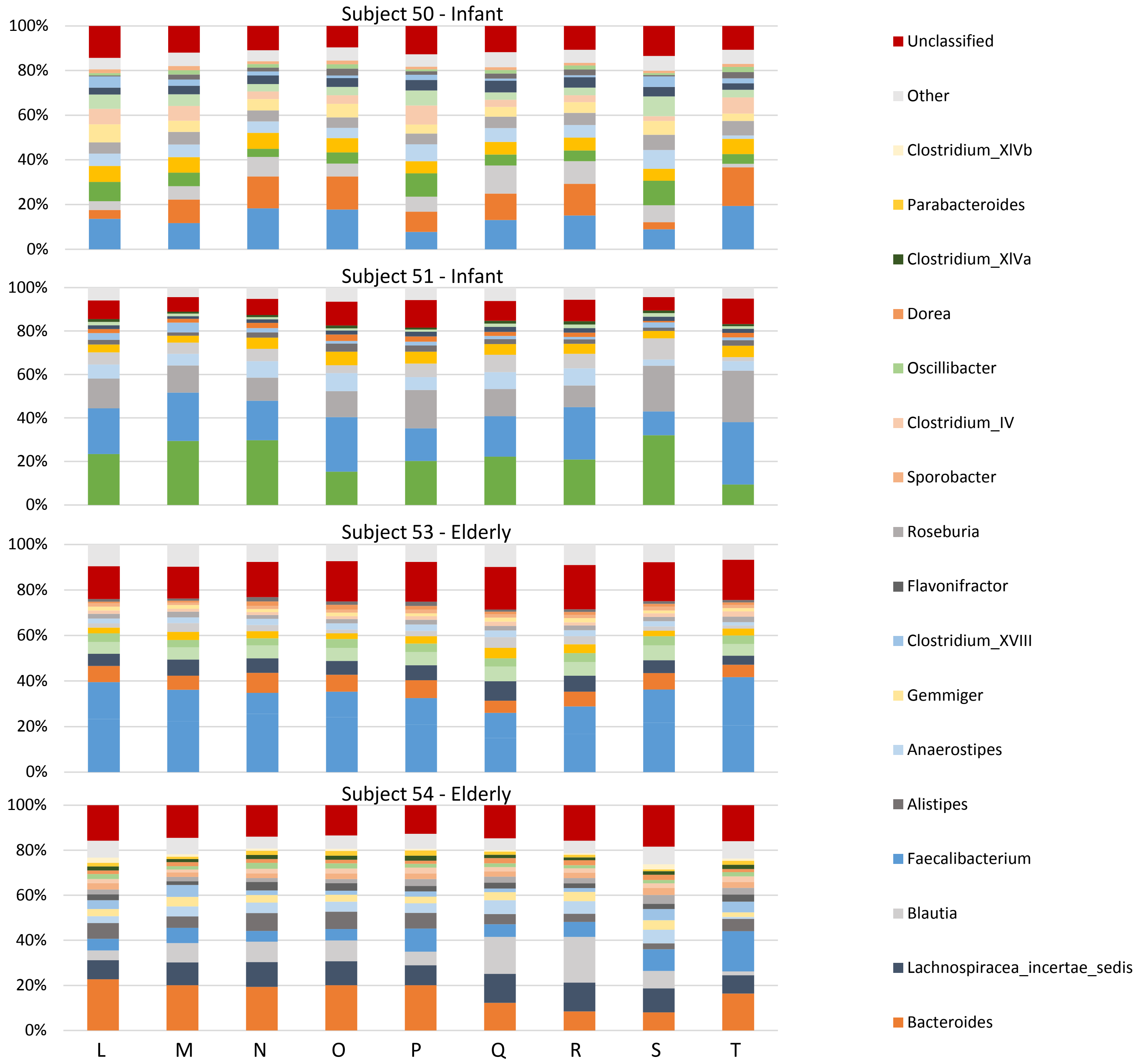

Subject 50 - Infant
Subject 51 - Infant
Subject 53 - Elderly
Subject 54 - Elderly
100%
80%
60%
40%
20%
0%
L
M
N
O
P
Q
R
S
T
Unclassified
Other
Clostridium_XlVb
Parabacteroides
Clostridium_XlVa
Dorea
Oscillibacter
Clostridium_IV
Sporobacter
Roseburia
Flavonifractor
Clostridium_XVIII
Gemmiger
Anaerostipes
Alistipes
Faecalibacterium
Blautia
Lachnospiracea_incertae_sedis
Bacteroides
