# Supplementary material for: Effect of room temperature transport vials on DNA quality and phylogenetic composition of faecal microbiota of elderly adults and infants
Source: Microbiome. 2016 May 10;4:19. doi: 10.1186/s40168-016-0164-3 (PMC4862223; doi:10.1186/s40168-016-0164-3)
Supplement: Additional file 4: Figure S3. — Majority of samples cluster by subject showing stability of microbiota composition after storage. A) Dendrogram using hierarchical clustering of a Spearman distance matrix, showing clustering of samples with their technical replicates. Subject 41 is highlighted as the only elderly subject where the samples did not cluster together B) Magnification to indicate infant subjects. Subjects 08, 09 and 16 are highlighted as subjects where samples do not cluster together. Infant subject 22 was extracted under nine difference conditions, and each condition was replicated by a second PCR. (PDF 245 kb) [file 40168_2016_164_MOESM4_ESM.pdf]

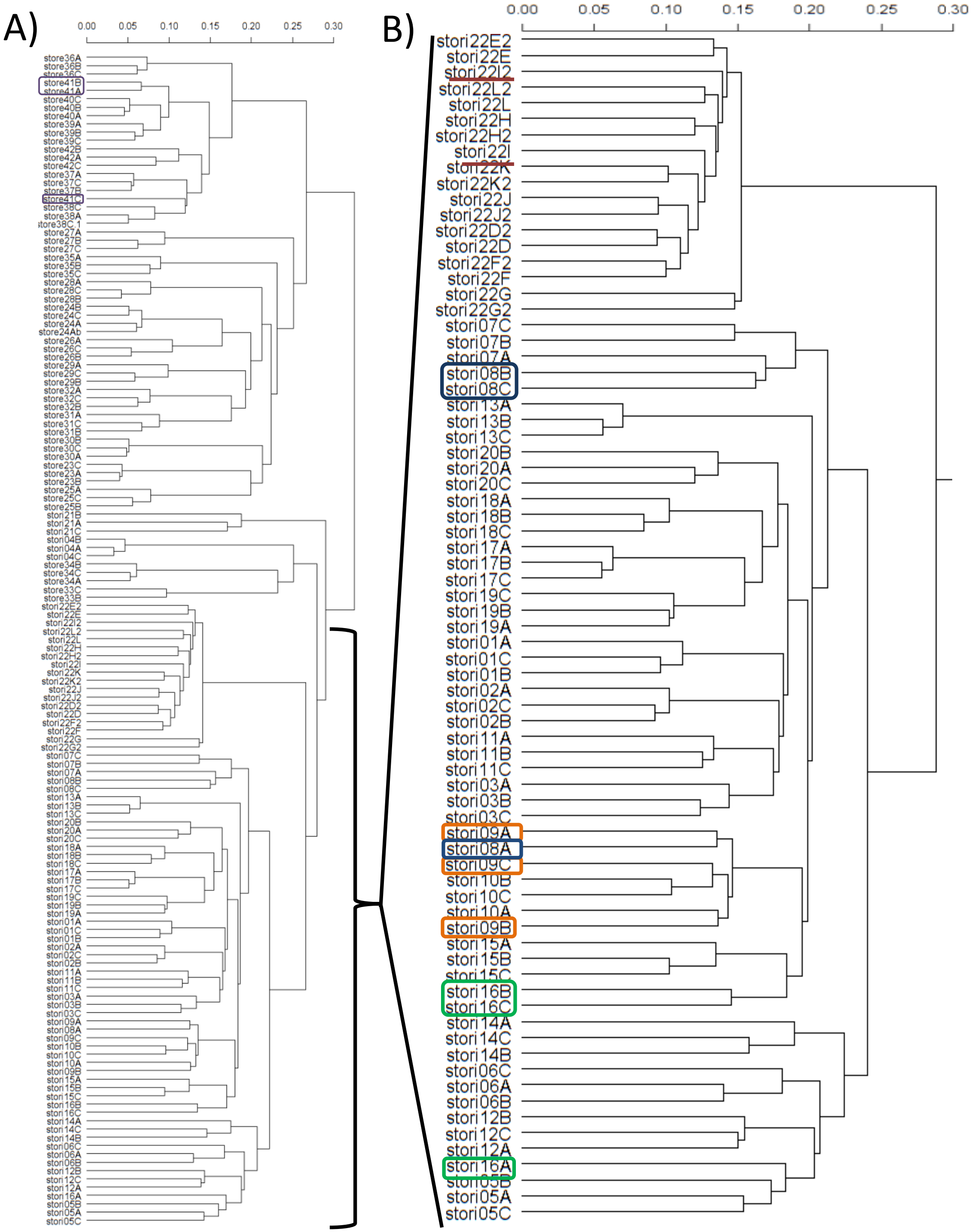

A)
0.00
0.05
0.10
0.15
0.20
0.25
0.30
B)
0.00
0.05
0.10
0.15
0.20
0.25
0.30
stori22E2
stori22E
stori22I2
stori22L2
stori22L
stori22H
stori22H2
stori22I
stori22K
stori22K2
stori22J
stori22J2
stori22D2
stori22D
stori22F2
stori22F
stori22G
stori22G2
stori07C
stori07B
stori07A
stori08B
stori08C
stori13A
stori13B
stori13C
stori20B
stori20A
stori20C
stori18A
stori18B
stori18C
stori17A
stori17B
stori17C
stori19C
stori19B
stori19A
stori01A
stori01C
stori01B
stori02A
stori02C
stori02B
stori11A
stori11B
stori11C
stori03A
stori03B
stori03C
stori09A
stori08A
stori09C
stori10B
stori10C
stori10A
stori09B
stori15A
stori15B
stori15C
stori16B
stori16C
stori14A
stori14C
stori14B
stori06C
stori06A
stori06B
stori12B
stori12C
stori12A
stori16A
stori05B
stori05A
stori05C
